# GABA: a key player of abiotic stress regulation

**DOI:** 10.1080/15592324.2022.2163343

**Published:** 2023-01-19

**Authors:** Vipul Mishra, Priya Gahlowt, Samiksha Singh, Nawal Kishore Dubey, Surendra Pratap Singh, Durgesh Kumar Tripathi, Vijay Pratap Singh

**Affiliations:** a Plant Physiology Laboratory, Department of Botany, C.M.P. Degree College, a Constituent Post Graduate College of University of Allahabad, Prayagraj, India; b Centre of Advanced Studies in Botany, Institute of Science, Banaras Hindu University, Varanasi, India; c Plant Molecular Biology Laboratory, Department of Botany, Dayanand Anglo-Vedic (PG) College, Chhatrapati Shahu Ji Maharaj University, Kanpur, India; d Crop Nanobiology and Molecular Stress Physiology Lab, Amity Institute of Organic Agriculture (AIOA), Amity University Uttar Pradesh, Noida, India; e Department of Botany, S.N. Sen B.V. P.G. College, Kanpur, India

**Keywords:** Aminobutyric acid (GABA), heat stress, light stress, stress acclimation, yield

## Abstract

Abiotic stress is considered as the main culprit for reduction of global food production. Recent studies have reported GABA as a major regulator of abiotic stress and thus opening new avenues in research on emerging roles of GABA in abiotic stress acclimation in plants.

A four-carbon well recognized, ubiquitous, non-proteinogenic amino acid, -aminobutyric acid (GABA) was initially discovered as a metabolite in plants during the mid-20^th^ century in potato tubers.^1^ But, the focus shifted to animals after the revelation of its function in neurotransmission, especially due to its role in calming anxiety,^2^ due to which recently CRISPR-edited and GABA-enriched tomato has been made available in the market.^3^ It has also been reported as an endogenous signaling molecule in the plants during their growth and developmental processes.^4^ Eventually, the research also paced for the elucidation of GABA’s function in plants after the finding of its active role in combating varying biotic and abiotic stress. Being an intermediate of nitrogen metabolism, GABA also contributes to primary and secondary metabolic pathways in plants.^5^ Several studies have provided evidences about the relationship of GABA and polyamines with different plant hormones such as abscisic acid, cytokinin, auxins, gibberellins and ethylene, etc., which are important for the plants facing different stress conditions. As per foregoing works, GABA has been found to be involved in enhancing the plant tolerance toward temperature,^6,7^ water scarcity,^8^ salinity,^9^ low light and nitrogen starvation.^10^ The role of GABA regarding single stress effect is sufficiently available with adequate researches, whereas its effects during combined stress on plants are not enough. Works on stress combinations of salt and drought, as well as salt, drought, and heat combined,^11^ are an example of GABA’s role in their regulation. One such experiment regarding combined stress has been performed by Balfagon et al.^12^ which is concomitant with GABA’s rescuing property but with different gene expressions in comparison to single stress subjection.

Though, GABA accumulates aggressively under stressed cellular milieu, it is essentially present in all the plants analyzed so far under optimum concentrations for proper plant growth. There are two known pathways of its endogenous biosynthesis, *viz*. the GABA shunt pathway and generation through the breakdown of polyamines (Pas).^12^

Balfagón et al.^12^ exposed *Arabidopsis thaliana* Col-0 and its mutants to elevated light intensity (HL), heat stress (HS) and their combination (HL+HS), as it is both the stresses are seldom separable in the environment. They did the relative analysis of stressors on mutants of GABA biosynthesis (*GLUTAMATE DESCARBOXYLASE* [*gad*] 3, i.e., *gad3* (SALK_138534C and SALK_033307C), *gad1-5* (CS860069)), and autophagy impaired mutants (autophagy-related proteins [*atg*] 5 and 9, i.e., *atg5* and *atg9)* in order to analyze the metabolic accumulates and degenerates, physiological responses and their mitigation by GABA in coordination with autophagy.

The authors interestingly reported that HL+HS facing Col-0 plants show an upsurge in the transpiration and stomatal conductance rather than stomatal closure related with minimizing loss of water during stress along with the limited rate of photosynthesis, suggestive of strategy to rescue antenna complex and simultaneously cooling down of the leaf temperature. Furthermore, the metabolite analysis using gas chromatography and TOF mass spectrometer specifically display a heightened increase in the level of GABA along with glycerol, succinic acid, rhamnose, arginine, gluconic acid, tyrosine, under HL+HS justifying its significance in mitigating amalgamated stress. Furthermore, glucose, fructose, maltose and raffinose get accumulated in HL+HS along with slight increase in sucrose, conclusive of the increased photosynthetic accumulates due to the increased energy demand and HL. Alternatively, the assay of the TCA cycle transcriptome and metabolome displays diminishing metabolite levels, particularly aspartate and glutamate with high rate of TCA-related enzymes expression, indicative of imbalance of TCA cycle where the throughput genic expression was high but with low functional metabolites.

In contrast to Col-0, the absence or declining of GABA is observed in *gad3* mutant lines, when examined under HL+HS followed by a sharp decline in the survival rate percentage and increased leaf damage index along with extremely low rate of photosynthesis, transpiring rate and stomatal conductance, and damaging increment in the leaf temperatures. These results established that GABA is intimately involved and essential for the rescue of plants stressed with HL+HS. While in the case of individual stressors either HL or HS, the presence of GABA remains unnoticeable. Using RNA sequencing data, analysis of transcript expression involves in GABA biosynthesis and GABA catabolism shows remarkable increase in *GAD1* and *GAD3* (the GABA biosynthetic transcripts), whereas there is decrease in expression of *POP2* and *ALDH5F1* (the GABA catabolic transcripts) observed in HL+HS. The minor recovery noticed in *gad3* may be subjected to the action and presence of *GAD1*, synthesizing GABA in miniscule amounts as *GAD1* displays a 4.64% and *GAD3* displays 29.03% enhancement in Col-0 under HL+HS. Reduced glutamate and putrescine levels signify the GABA generation through both its biosynthetic pathways, i.e., GABA shunt and oxidation of polyamines. Abiotic stress causes putrescine oxidation which results in GABA production; this signifies that reduction in putrescine level during combined stress causes accumulation of GABA in plants.

Interestingly, a similar observation is to be made for the phenomenon of autophagy, where Col-0 plants show enhanced resistance to HL+HS in comparison to *atg*(5 and 9) mutants while survival rate under HL or HS continued to be nearly unaffected in *atg* plants showing minor leaf damage but not death under HL. Along with the recent reports, this result overlaps with the fact that the triggers of GABA and autophagy-related genes under HL+HS might be interlinked and tend to acclimatize the plant against stress by decreasing the leaf temperatures through stomatal conductance and increasing transpiration thereby suppressing the leaf death index.

In conclusion, the idea of studying two interrelated stresses and combining them makes the experimental setup closer to the natural growth conditions. HL+HS stress combination displays morphological, physiological and metabolomic variations with respect to individual HL or HS, with GABA acting as a rescuer of them. Exogenous application of GABA has been reported to improve the plant tolerance to different stresses. It is being evident that GABA enhances expression of certain heat shock factors, causing the plants to become heat tolerable.^13^ It would be interesting to study the role of GABA biosynthesis (*GAD1, GAD2, GAD3*, and *GAD)*, GABA catabolism transcripts (*ALDEHYDE DEHYDROGENASE 5F1* [*ALDH5F1*] and *POLLEN-PISTIL INCOMPATIBILITY 2* [*POP2*])^12,14^ expression under different combination of interrelated stresses of nature, like drought often accompanied with high heat and high light (Figure 1). Drought further affects the plant growth by limiting the transpiration and stomatal conductance which is similar to HL but unlikely of HL+HS. Also, *GAD1/2* has been seen to function in rescue utilizing GABA under drought^15^ and salinity stress.^16^ Therefore, it would be intriguing to see which one of the known transcripts and metabolites get involved in such stress combination as reported by Balfagón et al. ^12^ and Shaar-Moshe et al.^11^ or if other unique transcripts partake during the process thereby bringing research closer to formulate strategies for better adapted plants against stress combination. Further work on amalgamated stress may provide various unknown aspects of GABA which are speculated to deal with abiotic stress in precise manner. It would be an interesting arena for the researchers to formulate strategies for the plants for their better adaptation to abiotic stresses on global level.

**Figure 1.**
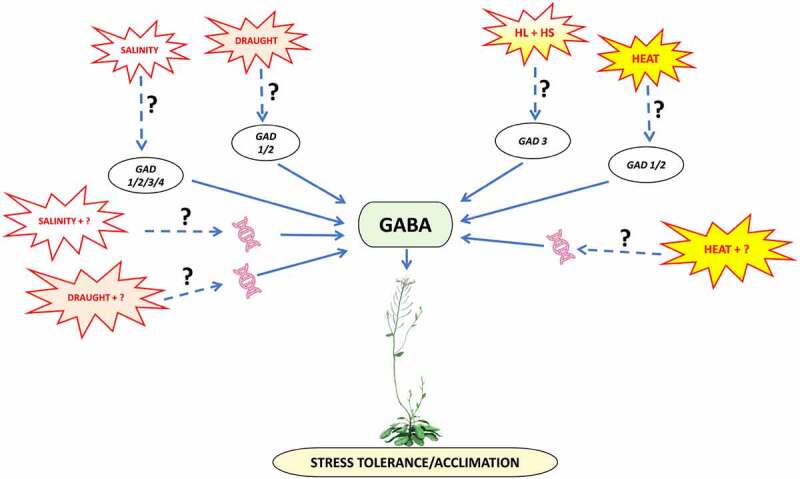
Schematic illustration of GABA being produced under the varying abiotic stresses through GABA shunt pathway utilizing the different GLUTAMATE DESCARBOXYLASE [GAD] transcripts to acclimatize the plant to stress. Different transcripts of GAD are expressed under stress combination (HL+HS) in comparison to single stress of the combination through unknown intermediaries. This difference paves way for stress tolerance *via* GABA synthesized through different GAD transcripts to be studied under other stress combinations too.
